# Clinically relevant fluoroquinolone resistance due to constitutive overexpression of the PatAB ABC transporter in *Streptococcus pneumoniae* is conferred by disruption of a transcriptional attenuator

**DOI:** 10.1093/jac/dku449

**Published:** 2014-11-18

**Authors:** Alison J. Baylay, Laura J. V. Piddock

**Affiliations:** Antimicrobials Research Group, School of Immunity and Infection, Institute of Microbiology and Infection and College of Medical and Dental Sciences, University of Birmingham, Birmingham B15 2TT, UK

**Keywords:** multidrug resistance, efflux, antimicrobial resistance mechanisms, regulation

## Abstract

**Objectives:**

Constitutive overexpression of *patAB* has been observed in several unrelated fluoroquinolone-resistant laboratory mutants and clinical isolates; therefore, we sought to identify the cause of this overexpression.

**Methods:**

Constitutive *patAB* overexpression in two clinical isolates and a laboratory-selected mutant was investigated using a whole-genome transformation approach. To determine the effect of the detected terminator mutations, the WT and mutated *patA* leader sequences were cloned upstream of a GFP reporter. Finally, mutation of the opposing base in the stem–loop structure was carried out.

**Results:**

We identified three novel mutations causing up-regulation of *patAB*. All three of these were located in the upstream region of *patA* and affected the same Rho-independent transcriptional terminator structure. Each mutation was predicted to destabilize the terminator stem–loop to a different degree, and there was a strong correlation between predicted terminator stability and *patAB* expression level. Using a GFP reporter of *patA* transcription, these terminator mutations led to increased transcription of a downstream gene. For one mutant sequence, terminator stability could be restored by mutation of the opposing base in the stem–loop structure, demonstrating that transcriptional suppression of *patAB* is mediated by the terminator stem–loop structure.

**Conclusions:**

This study showed that a mutation in a Rho-independent transcriptional terminator structure confers overexpression of *patAB* and fluoroquinolone resistance. Understanding how levels of the PatAB efflux pump are regulated increases our knowledge of pneumococcal biology and how the pneumococcus can respond to various stresses, including antimicrobials.

## Introduction

*Streptococcus pneumoniae* is an important human pathogen representing a major disease burden worldwide. It is the main bacterial cause of community-acquired pneumonia, as well as being the cause of severe invasive infections such as meningitis. Fluoroquinolones are one of the classes of antibiotics frequently used to treat pneumococcal disease.^1^ However, fluoroquinolone resistance in pneumococci is easily selected and causes treatment failures.^2^ Known mechanisms of fluoroquinolone resistance in pneumococci are target site mutations in topoisomerase genes^3,4^ and the overexpression of the ABC transporter genes *patA* and *patB*.^5–7^ PatA and PatB dimerize to form a functional ABC transporter that exports certain hydrophilic fluoroquinolones, as well as other toxic compounds, such as the dye ethidium bromide.^8^

As well as drug efflux, PatAB has also been implicated in relieving a fitness cost associated with linezolid resistance mutations^9^ and intolerance of pH stress.^10^ Up-regulation of *patAB* has also been observed as the defining characteristic of a meningitis isolate compared with a bloodstream isolate taken from the same patient.^11^ These observations hint at a more fundamental role for the *patAB* transporter in pneumococcal stress responses. Elucidating the regulatory mechanisms controlling *patAB* may provide further insight into the role of this transporter in the pneumococcal cell. Specific regulators controlling expression of *patAB* are unknown, but two previous studies have suggested that expression of these genes is induced by exposure to sub-inhibitory concentrations of fluoroquinolones and by DNA damage.^12,13^

In two independent studies, constitutive overexpression of *patAB* was associated with a G–T mutation 46 bp upstream of *patA*.^9–11^ This is predicted to affect folding of a Rho-independent transcriptional terminator, and hypothesized to allow increased transcriptional read-through into *patA*.^11^ In this study, we identified three novel mutations affecting this putative terminator structure in fluoroquinolone-resistant clinical isolates of *S. pneumoniae* with constitutive overexpression of *patAB*. We investigated the effect of these mutations on *patA* transcription using GFP reporter fusions, and showed that transcriptional repression of GFP could be restored by reconstituting the terminator base-pairing structure. This study shows that attenuator mutations mediate clinically relevant overexpression of *patA(B)* and fluoroquinolone resistance.

## Materials and methods

### Bacterial strains and growth

All *S. pneumoniae* strains and clinical isolates (Table 1) were grown statically at 37°C in an atmosphere of 5% CO_2_ in brain heart infusion broth (BHI; Oxoid, Basingstoke, UK) or on Columbia agar (Oxoid, Basingstoke, UK) supplemented with 5% defibrinated horse blood (TCS Biosciences, Buckingham, UK). Bacterial growth in broth was assessed by measurement of OD at 660 nm (OD_660_) of liquid cultures. *Escherichia coli* strains used for plasmid construction were grown overnight on LB (Oxoid, Basingstoke, UK) agar at 37°C or in LB broth at 37°C with shaking at 180 rpm.

**Table 1.** Bacterial strains used in this study

Strain	Description	Reference
R6	unencapsulated WT strain, ATCC BAA255	27
M168	reserpine-resistant mutant derived from M4	7
M184	reserpine-resistant mutant derived from R6	7
M87	clinical isolate from MRL Pharmaceutical Services	28
M101	clinical isolate from MRL Pharmaceutical Services	28
M505	R6 recipient transformed with M101 DNA, selected on 8 mg/L ethidium bromide	this study
M506	R6 recipient transformed with M187 DNA, selected on 8 mg/L ethidium bromide	this study
M507	R6 recipient transformed with M174 DNA, selected on 8 mg/L ethidium bromide	this study
M511	R6 containing pBAV1K-gfp82 vector	this study
M512	R6 containing construct pBAV1K-gfp82-WT	this study
M513	R6 containing construct pBAV1K-gfp82-v101	this study
M514	R6 containing construct pBAV1K-gfp82-v101c	this study
M517	R6 containing construct pBAV1K-gfp82-v168	this study
M518	R6 containing construct pBAV1K-gfp82-v168c	this study
M515	R6 containing construct pBAV1K-gfp82-v87	this study
M516	R6 containing construct pBAV1K-gfp82-v87c	this study

### DNA extraction and PCR

Genomic DNA was extracted from all strains using the Wizard Genomic DNA extraction kit (Promega) following the manufacturer's instructions. The primer sequences and PCR conditions used in this study are listed in Table 2.

**Table 2.** Primers and DNA oligonucleotide used in this study

	Target	Forward primer	Reverse primer
Primer pair			
1	*patA*	TCTTGCTCAGTCCATCATCGAA-TATA	CCGCTGTGGATTAGTTCATTTCC
2	*patB*	AGAATCCAGTCCAGCGAAAGCT	GAAAGAACGACCAGATGTTCCAAT
3	*patA* first half	TCTTGCTCAGTCCATCATCGAA-TATA	CAGCATCGGTTCCTTGTC
4	*patA* second half	CAGATGAAGAGTTGGTTGGA	CCGCTGTGGATTAGTTCATTTCC
5	*patA* promoter	GATAGGGCAGAAGAGCATCC	GATAACGCGGTTGCAGAAGT
6	*patA* qRT–PCR	TCTTAGGCGCCCTCCTTACT	ATAGGCTGCGAGGACAAC
7	*patB* qRT–PCR	AGAAATGTGACGCTGGCTCT	TTCTGCTGGAGGTTGGTGT
8	*guaA* qRT–PCR	GCGCTTCGTCAGAATAAACC	AGTCCTTGCCAGTGACCTTC
9	spr1886 qRT–PCR	GGATTGGGAATCGTTTAGGG	AGAATCCAGTCCAGCGAAAG
10	*hexA* qRT–PCR	TGTCTAGTGTGCCACGGATT	CGCTGCGCTAATCAAACTCT
11	PBAV1K-gfp82 cloning site	TAGTATCGACGGAGCCGATT	TGTGCCCATTAACATCACCA
DNA oligonucleotide			
WT *patA* upstream region	taa**gaattc**aaccaagactcactagttaatctagctgtatcaaggagacttctttgacaattctccacttttttgcta gaataacatcacacaaacagaatgaaaaggagctgacgcattgtcgctcccttttgtctattttt**tctaga**aag		

Bold and underlined text represents restriction enzyme cleavage sites.

### Transformation

Transformation of *S. pneumoniae* R6 was carried out as described previously.^7^ Briefly, mid-logarithmic phase cultures of R6 were diluted 1 : 20 in competence medium [Todd–Hewitt broth (Oxoid, Basingstoke, UK) containing 1 mM calcium chloride (Sigma Aldrich Ltd, Dorset, UK), 0.2% BSA (Sigma Aldrich Ltd, Dorset, UK) and 100 ng/mL competence-stimulating peptide 1 (CSP1; Mimotopes, Clayton, Victoria, Australia)]. For whole-genome transformation, genomic DNA from the donor strain was added to a final concentration of 0.2 mg/L to aliquots of the competent cell suspension. For transformation with PCR amplimers, 20 μL of purified PCR amplimer was added to 500 μL of cell suspension. Transformation reactions were incubated for 3 h at 37°C, then 20 and 200 volumes were spread onto agar plates containing 8 mg/L ethidium bromide. Viable counts were determined in parallel to allow estimation of the transformation frequency.

### Illumina sequencing and data analysis

Illumina whole-genome sequencing was carried out by Genepool (Edinburgh, UK) according to standard protocols to give 100 bp paired-end reads. Reads were mapped against the published R6 genome sequence (GenBank accession number NC_003098) using Bowtie2^14^ using the –very-sensitive-local setting, and a consensus pileup was generated using mpileup in Samtools (version 0.1.18).^15^ Sets of putative SNPs and small indels were generated using BCFtools (version 0.1.17-dev).^15^

### Susceptibility of S. pneumoniae to antibiotics and dyes

The MICs of ciprofloxacin, norfloxacin and ethidium bromide were determined using the standardized agar doubling dilution method according to the BSAC.^16^

### Ethidium bromide accumulation

Intracellular accumulation of ethidium bromide was measured by monitoring fluorescence over time, as follows. Logarithmic-phase bacterial cultures were pelleted by centrifugation at 2200 **g** and resuspended in PBS (Life Technologies, cat. no. 14040-133; 2160 mg/L Na_2_HPO_4_, 8000 mg/L NaCl, 200 mg/L KH_2_PO_4_, 200 mg/L KCl, 100 mg/L MgCl_2_ and 100 mg/L CaCl_2_). Cell suspensions were adjusted to an OD_660_ of 0.1 with fresh PBS, and a 100-cell suspension was added in triplicate to a black microtitre tray. Fluorescence was measured at excitation and emission wavelengths of 530 and 600 nm, respectively, using a FLUOstar Optima plate reader (BMG Labtech, Aylesbury, UK) every 2 min for a total of 30 min. A final concentration of 100 μM ethidium bromide was added to each well by injection on the second cycle of measurement.

### qRT–PCR

Total RNA was extracted from three biological replicates of logarithmic-phase cultures of each test strain using a Promega SV Total RNA Isolation kit according to the manufacturer's instructions (Promega, Southampton, UK). RNA and contaminating DNA concentrations were measured using a Qubit fluorimeter according to the manufacturer's instructions (Life Technologies, Paisley, UK). Residual DNA contamination was removed by treatment with Turbo DNase (Life Technologies), and cDNA was generated using Superscript III reverse transcriptase (Life Technologies) following the first-strand synthesis protocol supplied by the manufacturer. Expression of *patA*, *patB*, spr1886 and *guaA* was measured relative to expression of *rpoB* by SYBR Green Quantitative real-time (qRT) PCR. Reactions consisted of 12.5 μL IQ SYBR Green Supermix (Bio-Rad, Hemel Hempstead, UK), 375 nM each of forward and reverse primers and 1 μL cDNA in a 25 µL reaction. Real-time PCR was carried out using a Bio-Rad CFX96 thermal cycler with the following protocol: 3 min at 95°C, followed by 40 cycles of 10 s at 95°C and 30 s at 54.5°C. Expression values were calculated using the Pfaffl method.^17^

### patA expression reporter plasmid construction and GFP assay

To generate plasmid pBAV1K-gfp82, plasmids pBAV1K-T5-gfp^18^ and pMW82^19^ were digested with EcoRI and PstI (Thermo Scientific) and digestion products separated by agarose gel electrophoresis. The 2800 bp plasmid backbone of pBAVK1K-T5-gfp and the 824 bp band corresponding to the *gfp* gene of pMW82 were extracted using a Qiagen gel extraction kit according to the manufacturer's instructions. The DNA fragments were ligated using QuickStick ligase (Bioline) and used to transform *E. coli* Top10 cells (Life Technologies). Transformants were selected on 50 mg/L kanamycin. To remove DNA methylation for subsequent digestions, extracted plasmids were transformed into *E. coli* JM110 cells and re-purified.

To clone the *patA* promoter into pBAV1K-gfp82, giving pBAV1K-gfp82-WT, a 144 bp DNA fragment was synthesized by GeneArt (Life Technologies) that covered the region from 12 to 146 bp upstream of the *patA* start codon and incorporated a 5′ EcoRI site and a 3′ XbaI site. The promoter fragment was digested with EcoRI and XbaI, purified using a QIAquick PCR purification kit (Qiagen) and combined in a 100 : 1 ratio with pBAV1K2 DNA linearized with XbaI and EcoRI. The DNA fragments were ligated with QuickStick ligase and used to transform *E. coli* Top10 cells. Transformants were selected on 50 mg/L kanamycin and screened for successful incorporation of the insert by PCR with primer pair 11.

Plasmid DNA was extracted from *E. coli* cells using a QIAquick Miniprep Kit according to the manufacturer's instructions, and 20 μL of each plasmid preparation was used to transform *S. pneumoniae* R6 as described above. Successful transformants were selected on plates containing 100 mg/L kanamycin.

To measure expression of *patA* from the reporter construct, stationary-phase cultures of R6 containing pBAV1K-gfp82 constructs were diluted to an OD_600_ of 0.01 in BHI broth, and 200 μL of diluted culture was added in duplicate to a black microtitre tray with a clear base (Corning). Cultures were incubated in a Fluostar Optima at 37°C for 12 h, and simultaneous fluorescence (excitation wavelength 492 nm, emission wavelength 520 nm) and absorbance (600 nm) measurements were taken every 3 min. Absorbance and fluorescence values at each timepoint were calculated as the average of three biological replicates.

### RNA structure prediction

Free energies of folding of RNA structures were predicted computationally using the RNAfold program from the Vienna RNA suite.^20^

## Results

### Genetic determinants causing fluoroquinolone resistance and patAB up-regulation could be transferred into an antibiotic-susceptible strain by a single round of transformation

Laboratory mutants and clinical isolates of *S. pneumoniae* that constitutively overexpress *patAB* have been described previously,^7,9,11,12,21^ but the mechanism has not been identified. Two recent whole-genome sequencing publications described a G–T mutation 46 bp upstream of *patA* associated with fluoroquinolone resistance.^9,11^ In this study, we investigated the cause of constitutive *patAB* overexpression in two further fluoroquinolone-resistant clinical isolates and a laboratory mutant. The two clinical isolates, M101 and M87, were chosen from a set of previously described clinical isolates with elevated *patAB* expression.^21,22^ The third strain investigated was M168, a laboratory-selected mutant of type strain NCTC7465, selected for resistance to the efflux inhibitor reserpine.^7^

In the case of the laboratory-selected mutants, mutations causing the desired phenotype could be identified by a whole-genome re-sequencing approach: sequencing of the mutant and detection of polymorphisms with respect to the genome sequence of an isogenic parental strain. However, for the two clinical isolates investigated in this study, antibiotic-susceptible parental strains were not available for comparison so this approach could not be used. However, viable transformants could be selected on ethidium bromide (8 mg/L), a known PatAB substrate, when the unencapsulated laboratory strain R6 was transformed with high-molecular weight DNA extracted from either M101 or M87 (giving R6^M101^ and R6^M87^, respectively). Although a parental strain was available for the laboratory-selected strain M168, the same approach of whole-genome transformation into R6 (giving R6^M168^) was followed for consistency.

In all three cases, a single round of transformation was sufficient to select transformants with an ethidium bromide MIC of 32 or 64 mg/L, three to four dilutions higher than that of R6. The ethidium bromide MICs were slightly higher than those for the donor strains, possibly reflecting the expression of *patAB* in a different genetic background.

**Figure 1.**
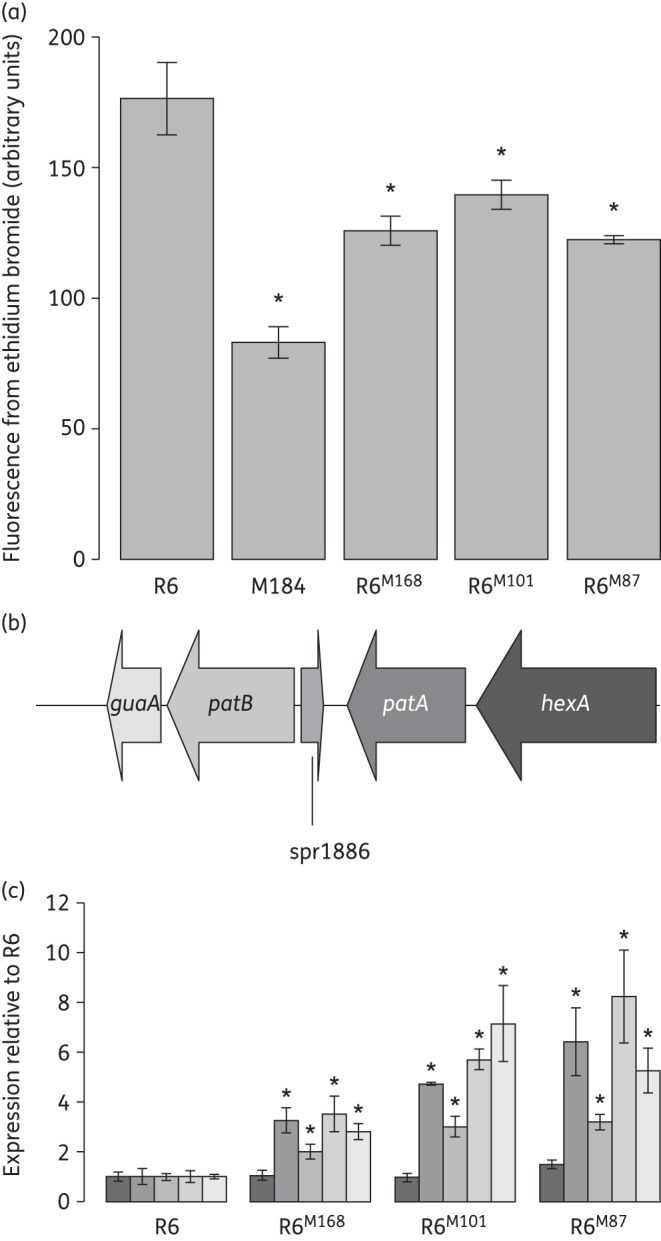
Confirmation of *patAB* overexpression phenotype in transformants R6^M168^, R6^M101^ and R6^M87^. (a) Fluorescence due to intracellular accumulation of ethidium bromide following 10 min of incubation with 100 μM ethidium bromide. Bars and error bars represent averages and standard deviations of three biological replicates. *Accumulation significantly lower than R6; *P *<* *0.05, one-tailed Student's *t*-test. A previously described strain, M184, was used as a positive control. (b) Representation of the *patAB* genetic locus. (c) Expression of *patA*, *patB* and neighbouring genes in R6 and transformants R6^M168^, R6^M101^ and R6^M87^ measured by qRT–PCR compared with *rpoB*. Bars represent average expressions relative to R6 from three biological replicates and are shaded by gene as in panel (b). Error bars represent standard deviations of three biological replicates. *Expression significantly different from R6; *P *<* *0.05, two-tailed Student's *t*-test.

To confirm that the selection for ethidium bromide resistance correlated with the transfer of mutations causing *patAB* overexpression, we carried out three checks. Firstly, antibiotic susceptibility profiles were determined by agar doubling dilution MIC (Table 3). All three transformants were less susceptible to ciprofloxacin, norfloxacin and ethidium bromide compared with R6, and this was reversed in the presence of sodium orthovanadate, an inhibitor of ABC transporters. The susceptibility of R6 to all three agents was also increased slightly in the presence of sodium orthovanadate, most likely due to inhibition of basal levels of the PatAB expressed by the R6 strain. Secondly, the intracellular accumulation of ethidium bromide was measured by fluorescence assay. Transformants showed a 20%–30% reduction in accumulation of ethidium bromide compared with R6 (Figure 1a). A previously described strain, M184,^7^ which expresses *patAB* at a very high level (>100-fold higher than R6, data not shown) was used as a positive control.

**Table 3.** MICs of PatAB substrates for transformants R6^M168^, R6^M101^ and R6^M87^

Strain	MIC (mg/L)					
	CIP	CIP + Ort	NOR	NOR + Ort	EtBr	EtBr + Ort
R6	1	0.5	4	1	4	<1
M184	**4**	0.5	**32**	2	**64**	<1
R6^M168^	2	0.5	**16**	2	**32**	<1
R6^M101^	**4**	0.5	**32**	2	**64**	<1
R6^M87^	**4**	0.5	**32**	2	**64**	<1

CIP, ciprofloxacin; NOR, norfloxacin; EtBr, ethidium bromide; Ort, 50 μM sodium orthovanadate.

Bold numbers indicate MIC values that are two or more dilutions higher than those for R6.

Finally, expression of *patA* and *patB* was measured directly by qRT–PCR. Expression of both *patA* and *patB* was significantly higher in R6^M168^, R6^M101^ and R6^M87^ than in R6 (Figure 1b and c and Table 4; *P *<* *0.05). Taken together, these results made us confident that mutations causing *patAB* overexpression had been successfully transferred using the whole-genome transformation approach.

**Table 4.** Expression of *patA* and *patB* in transformants R6^M168^, R6^M101^ and R6^M87^ compared with R6

	Fold change in expression compared with R6			
	*patA*		*patB*	
	average	SD	average	SD
R6^M168^	3.3	0.5	3.5	0.7
R6^M101^	4.7	0.1	5.7	0.4
R6^M87^	6.4	1.4	8.2	1.9

SD, standard deviation of three biological replicates.

### Sequencing of transformants revealed mutations in a Rho-independent terminator upstream of patA

**Figure 2.**
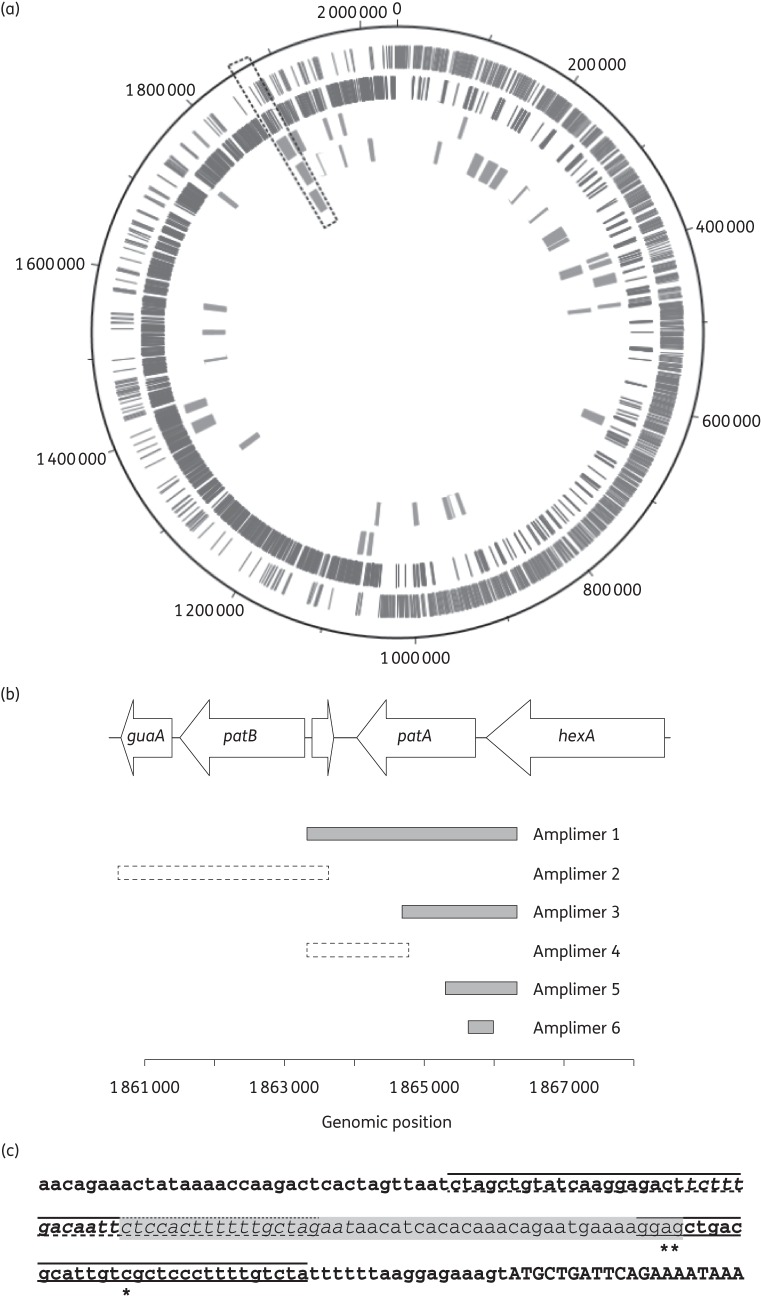
Locations of putative recombinant regions in transformants identified by whole-genome sequencing. (a) Recombinations mapped onto the R6 genome. Rings are numbered from outside to inside. Rings 1 and 2 represent R6 coding sequences encoded on the plus and minus strand, respectively. Rings 3, 4 and 5 represent putative recombinant regions in R6^M101^, R6^M87^ and R6^M168^, respectively. The region containing the *patA* and *patB* genes is indicated by the dashed box. (b) Extents of PCR amplimers from R6^M101^, R6^M87^ and R6^M168^ used for targeted transformation of R6 cells to determine the location of mutations causing *patAB* overexpression. Solid, filled boxes represent amplimers that produced ethidium bromide-resistant transformants. Dashed boxes represent amplimers for which no ethidium bromide-resistant transformants were obtained. (c) DNA sequence upstream of *patA*, with locations of mutations identified in R6^M101^, R6^M87^ and R6^M168^ indicated (*). The transcriptional attenuator predicted by Ribex, consisting of terminator (solid lines), anti-terminator (shaded text) and anti-anti-terminator (dashed lines) loops, is also indicated. The start of the *patA* coding sequence is shown in capital letters.

To identify mutations causing *patAB* overexpression in the three transformants, whole genomic DNA was extracted from strains R6, R6^M168^, R6^M101^ and R6^M74^ and sequenced using Illumina technology. Reads were aligned to the published R6 genome sequence (NC_003098) and SNPs detected. The laboratory stock of R6 used for our experiments differed from the published R6 genome sequence by 58 mutations (data not shown). These mutations were also observed in the three transformants and were discarded from further analysis. Regions of the transformant genomes where recombination had occurred between the donor and recipient genomes could be identified as clusters of SNPs relative to R6. Clusters were loosely defined as regions of the genome where the number of SNPs in a 5 kb sliding window was consistently greater than two. A single recombination event was observed in R6^M168^, occurring in the region of the genome that contains *patAB*. Multiple recombination events were observed in R6^M101^ and R6^M87^ (14 and 24, respectively). In both of these strains, however, there was a recombinant region in the vicinity of the *patA* and *patB* genes, as observed in R6^M168^ (Figure 2a).

The recombinant regions that encompassed the *patAB* genes were examined in more detail. The minimum sizes of the recombinant regions (naively defined as the distance between the first and last SNP in the cluster) ranged between 9.7 and 12.2 kb. The clusters consisted of 70 SNPs in R6^M168^, 64 in R6^M101^ and 62 in R6^M87^. To distinguish mutations causing *patAB* overexpression from natural polymorphisms between the donor strains and the recipient, we transformed R6 with targeted PCR amplimers from this region in M168, M101 and M87, and selected for ethidium bromide-resistant transformants (Figure 2b). We focused on the *patA* and *patB* genes themselves due to the considerable overlap of non-contiguous recombinant regions covering these genes in the three transformants. We obtained ethidium bromide-resistant transformants using an amplimer corresponding to the *patA* gene from all three donor strains. No ethidium bromide-resistant transformants were obtained with amplimers corresponding to patB. Transformation with further PCR amplimers corresponding to smaller segments of *patA* localized the mutations conferring ethidium bromide resistance to a region between genomic positions 1* *865* *645 and 1* *865* *963, corresponding to the intergenic region between *patA* and the upstream gene *hexA* in all three donor strains.

The region identified was examined in more detail. In each transformant, the identified region contained one SNP relative to R6. Relative to the start codon of *patA*, these were C(−33)T in R6^M168^, A(−47)C in R6^M101^ and G(−46)A in R6^M87^. A putative attenuator structure, consisting of a Rho-independent terminator, anti-terminator and anti-anti-terminator, was predicted upstream of *patA* using the Ribex server.^22^ The three identified mutations were all located within the terminator loop of this putative structure (Figure 2c). The mutation found in R6^M87^ occurred in the same position as the G–T mutation identified in the two previous studies;^9,11^ however, the nucleotide substitution in our strain was G–A.

### Identified mutations are predicted to reduce the stability of the Rho-independent terminator by disrupting base pairing

**Figure 3.**
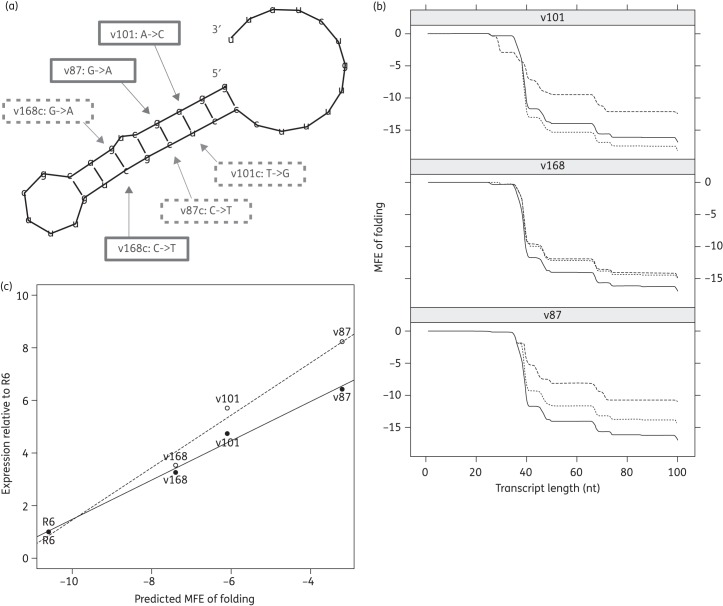
Structure of Rho-independent transcriptional terminator predicted by the RNAfold program from the Vienna RNA suite. (a) Predicted terminator structure. Mutations incorporated into pBAV1K-gfp82 constructs v101, v168 and v87 are indicated by solid boxes. Further mutations introduced in constructs v101c, v168c and v87c to attempt to restore base pairing are indicated by dashed boxes. (b) Folding energy of nascent *patA* transcript, predicted by RNAfold for WT (solid lines), mutant (v101, v168 and v87; dashed lines) and complemented (v101c, v168c and v87c; dotted lines) leader sequences. The nascent transcript was extended stepwise from the predicted *patA* transcription start site 67 bp upstream of the *patA* start codon. (c) Correlation between predicted stability of the *patA* transcript at 67 nt and expression of *patA* (solid line) and *patB* (dashed line) relative to R6 as measured by qRT–PCR.

The G–T mutation identified previously was predicted to reduce the stability of terminator loop folding, leading to increased transcription of the downstream gene. To predict whether our mutations were likely to have the same effect, we used the RNAfold program from the Vienna RNA suite to predict the RNA structure (Figure 3a) and determine the folding energy of WT and mutated terminator structures (Figure 3b). Introduction of any of the three mutations identified in our transformants increased the predicted free energy of folding of the terminator structure relative to WT (Figure 3b). The different mutations were predicted to destabilize the terminator to differing degrees. There was a strong positive correlation between predicted folding energy of the terminator and levels of expression of both *patA* (*R*^2^ = 0.98) and *patB* (*R*^2^ = 0.98) as measured by qRT–PCR (Figure 3c).

### Mutations disrupting terminator structure lead to increased transcription of a downstream GFP reporter

**Figure 4.**
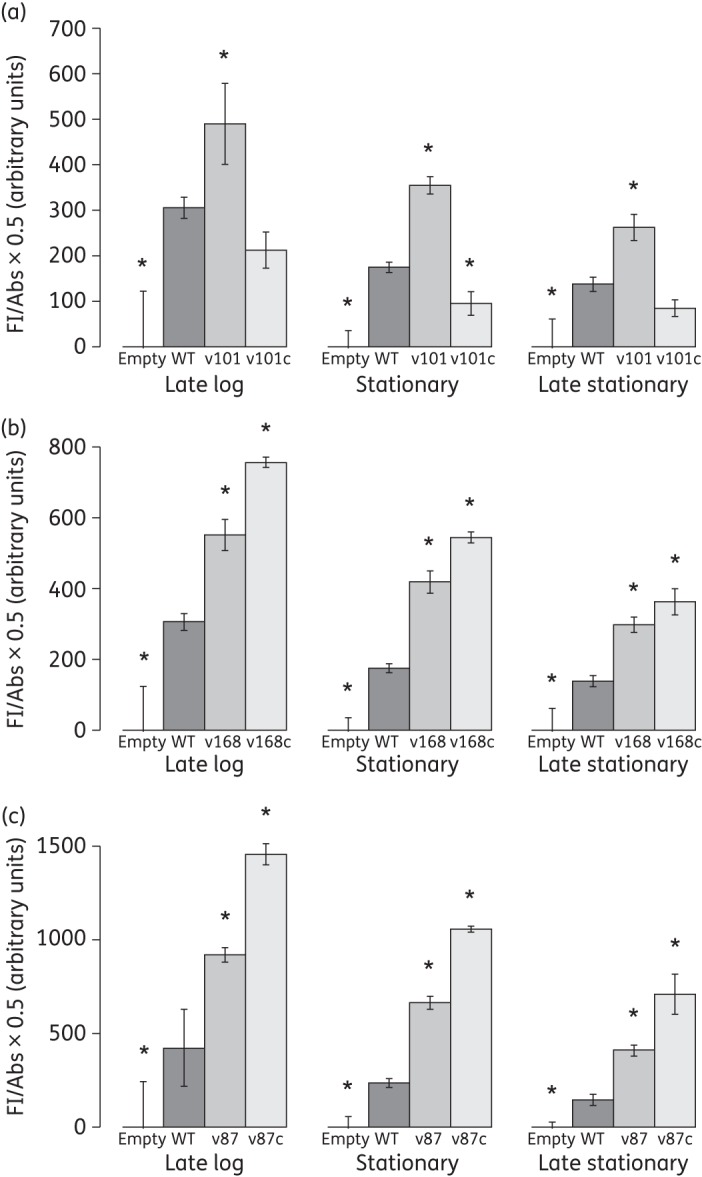
Fluorescence measured from pBAV1K-gfp82 reporter plasmid containing WT and mutant *patA* leader sequences during late-log (2.5 h), stationary (3.5 h) and late-stationary (5.5 h) phases of growth. Bars represent the fluorescence of pBAV1K-gfp82-WT and (a) v101 and v101c, (b) v168 and v168c and (c) v87 and v87c, adjusted to a standard OD_600_ of 0.5, relative to the fluorescence of empty pBAV1K-gfp82 vector. Error bars represent the standard deviation of three biological replicates. *Fluorescence significantly different from WT; *P *<* *0.05, two-tailed Student's *t*-test.

It was predicted that reduced stability of the terminator loop would increase transcription of *patAB*, by increasing transcriptional read-through into the *patA* coding sequence.^11^ However, this had not been experimentally demonstrated. To investigate this, we synthesized four DNA fragments corresponding to the *patA* upstream region (−146 to −13 bp), flanked by restriction sites. One fragment corresponded to the WT (R6) sequence, and the remaining three introduced the mutations C(−33)T (v168), A(−47)C (v101) and G(−46)A (v87). These were cloned upstream of a promoter-less GFP allele in a plasmid named pBAV1K-gfp82, derived from pBAV1K-t5-gfp (construction described in the Materials and methods section). The resulting constructs were transformed into R6 cells and transformants selected using 100 mg/L kanamycin. Fluorescence from each construct was measured during growth of the strains in BHI broth. Compared with a strain carrying pBAV1K-gfp82 empty vector, the pBAV1K-gfp82-WT construct produced consistently higher fluorescence throughout the growth cycle (Figure 4). Introduction of the terminator mutations (constructs pBAV1K-gfp82-v168, pBAV1K-gfp82-v101 and pBAV1K-gfp82-v87) resulted in a significant 1.5- to 3-fold increase in fluorescence in all three cases after 2.5, 3.5 and 5.5 h of growth, respectively, representing late-log, stationary and late-stationary phases, respectively (Figure 4; *P *<* *0.05, two-tailed Student's *t*-test).

### Restoration of terminator stem–loop stability leads to a reduction in transcription of the downstream reporter

The mutations investigated were predicted to cause *patAB* overexpression by disrupting the base pairing of the terminator stem–loop structure. Restoration of this base pairing should therefore restore termination and reduce transcription of the downstream gene. To investigate this prediction, for each mutant terminator we synthesized a further DNA fragment in which we attempted to re-form the predicted WT terminator structure by mutating the opposing base in the stem–loop such that Watson−Crick base pairing was restored (Figure 3a). This gave constructs pBAV1K-gfp82-v168c [C(−33)T and G(−43)A], pBAV1K-gfp82-v101c [A(−47)C and T(−30)G] and pBAV1K-gfp82-v87c [G(−46)A and C(−31)T]. In the case of v101c, mutating the opposing base in the structure resulted in a reduction of the predicted folding energy of the terminator (determined by RNAfold) back to below WT levels (Figure 3b). However, this was not the case for v168c and v87c, where mutation of the opposing base did not substantially reduce folding energy. Our model of the RNA folding was very simplistic and there may be further stabilizing interactions beyond simple base pairing that we did not take into account. Consistent with the predictions of folding energy, GFP fluorescence from pBAV1K-gfp82-v101c was significantly reduced compared with pBAV1K-gfp82-v101 at all tested growth stages (Figure 4a; *P *<* *0.05), and instead was either the same as or significantly lower than WT fluorescence. Fluorescence from pBAV1K-gfp82-v168c and pBAV1K-gfp82-v87c was not reduced by the attempt to restore base pairing, and remained significantly higher than WT at all tested growth stages (Figure 4b and c; *P *<* *0.05).

## Discussion

In this study, we have identified three novel SNPs causing up-regulation of *patAB* in *S. pneumoniae*. All three mutations were located in a Rho-independent transcriptional terminator located directly upstream of *patA*. Combined with the G−T mutation identified in two previous studies,^9,11^ four mutations have now been observed in this terminator structure, providing strong support for the hypothesis that this terminator structure is important for suppression of *patAB* expression and consequent fluoroquinolone resistance.

Rho-independent terminators consist of an RNA stem–loop structure that arrests progression of the transcription complex, followed by a uracil-rich region that promotes dissociation of the polymerase from the DNA.^23^ Mutations disrupting the stem–loop structure are therefore predicted to reduce polymerase pausing, and therefore dissociation, promoting continued transcription of the downstream gene. This hypothesis was previously proposed to explain the observed increase in *patAB* expression in terminator mutants;^11^ however, this had not been experimentally demonstrated. Here, we have used transcriptional fusion of the *patA* upstream region with a GFP reporter to demonstrate that terminator mutations do lead to increased transcriptional read-through into a downstream gene.

Fluorescence from the pBAV1K-gfp82-WT construct was consistently higher than from the empty vector at all growth stages tested. This suggests that *patAB* is transcribed at a basal level throughout growth. Disruption of the terminator stem–loop by all three mutations tested then increased this further. Two observations indicated that the stem–loop structure is important for transcriptional control. Firstly, there was a very strong correlation between predicted minimum free energy of the WT and mutant terminator structures and *patAB* expression level. Secondly, restoration of the stem–loop structure in the v101 variant by mutation of the opposing base in the structure reduced fluorescence back to WT levels. Taken together, these results suggest that the mutations increase downstream gene expression by reducing Rho-independent termination, as opposed to another mechanism, such as mutation of a transcription factor binding site.

It has previously been observed that isolates overexpressing *patAB* can be selected easily by exposure to the fluoroquinolone antibiotic ciprofloxacin or the efflux inhibitor reserpine.^7,24^ As demonstrated here, *patAB* overexpression can be conferred by any one of a number of mutations that disrupt the upstream terminator, which may explain how *patAB*-overexpressing mutants can be selected so reliably.

Disruption of the *patA* upstream terminator provides an explanation for selection of constitutive *patAB* expression on exposure to antibacterial agents. However, it does not explain the inducible *patAB* expression on exposure to fluoroquinolones or DNA-damaging agents that has been previously observed. Here, we observed that the Rho-independent transcriptional terminator is predicted to be part of a transcriptional attenuator, consisting of terminator, anti-terminator and anti-anti-terminator stem–loops. Transcriptional attenuation is a prevalent regulatory strategy in Gram-positive bacteria.^25^ Transcriptional attenuators are RNA leader sequences that can fold into two or more mutually exclusive stem–loops, one of which is a Rho-independent terminator, and this folding can be influenced by a variety of regulatory signals.^26^ Although it was not investigated in this study, it is conceivable that the presence of fluoroquinolones or DNA damage may result in induction of *patAB* expression via a regulatory signal that reversibly disrupts the *patA* upstream terminator. Recently, a similar transcriptional attenuator was found to be involved in up-regulation of the BmrCD ABC transporter in *Bacillus subtilis* in response to ribosome-targeting antibiotics.^27^ Similarly, expression of another *B. subtilis* ABC transporter gene associated with antibiotic resistance, *bmrA*, has been shown to be increased by an upstream mutation that increases mRNA stability.^28^ When combined with our results, these findings raise the intriguing possibility that transcriptional attenuation may be a common method of control of ABC transporter family efflux pumps in Gram-positive bacteria.

In this study, we located the mutations causing *patAB* overexpression using a whole-genome transformation approach that exploited the natural transforming ability of *S. pneumoniae*. This approach was similar to that used by Billal *et al*.^9^ to separate mutations causing linezolid resistance from bystander mutations. We used natural polymorphisms between the donor strains and the R6 recipient as a marker to distinguish regions of the genome that have undergone recombination, and therefore may contain mutations of interest. As a by-product of this, we observed primary and secondary recombinations in the same cell, as recently described by Croucher *et al*.^11^ We also saw multiple contiguous recombinations from the same piece of DNA, as was also described by Croucher *et al*.^11^ In this study, all three mutations were ultimately found to be located in the upstream region of *patA*, so they could have been discovered by other methods. However, the method used here has the potential to locate causative mutations anywhere in the genome, such as in a regulator encoded elsewhere. Due to recent improvements in whole-genome sequencing technology, meaning that many bacterial strains can be multiplexed in a single Illumina lane for a relatively low cost, this method could therefore be used to screen a larger library of *patAB*-overexpressing isolates to identify other components of the *patAB* regulatory pathway.

## Funding

This work was supported by a Medical Research Council Doctoral Training Grant (grant number DKAA GAS0025) to L. J. V. P.

## Transparency declarations

None to declare.
